# Risk of Mortality and Cardiovascular Events in Patients with Chronic Obstructive Pulmonary Disease Treated with Azithromycin, Roxithromycin, Clarithromycin, and Amoxicillin

**DOI:** 10.3390/jcm13071987

**Published:** 2024-03-29

**Authors:** Imane Achir Alispahic, Josefin Eklöf, Pradeesh Sivapalan, Alexander Ryder Jordan, Zitta Barrella Harboe, Tor Biering-Sørensen, Jens-Ulrik Stæhr Jensen

**Affiliations:** 1 Department of Internal Medicine, Respiratory Medicine Section, Herlev and Gentofte Hospital, University Hospital of Copenhagen, 2100 Copenhagen, Denmark; josefin.viktoria.ekloef@regionh.dk (J.E.); pradeesh.sivapalan.02@regionh.dk (P.S.); jens.ulrik.jensen@regionh.dk (J.-U.S.J.); 2 Department of Respiratory and Infectious Diseases, Copenhagen University Hospital, 3400 North Zealand, Denmark; zitta.barrella.harboe@regionh.dk; 3 Department of Cardiology, Gentofte University Hospital, 2900 Hellerup, Denmark; tor.biering-soerensen@regionh.dk; 4 Department of Clinical Medicine, University of Copenhagen, 2200 Copenhagen, Denmark

**Keywords:** COPD, major adverse cardiovascular event, stroke, AMI, cardiovascular death, clarithromycin, azithromycin, roxithromycin, amoxicillin

## Abstract

**Background:** Prior research has raised concerns regarding the use of macrolides and their association with an increased risk of cardiovascular events. **Methods:** We conducted a cohort study, where we explored the cardiovascular risks associated with the treatment of COPD patients using macrolide antibiotics–namely azithromycin, clarithromycin, and roxithromycin—with amoxicillin serving as a reference. The study focused on COPD patients in an outpatient setting and included a thorough 3-year follow-up. Patients were categorized into four groups based on their treatment. The primary analysis utilized an adjusted Cox model, supplemented by sensitivity analysis through inverse probability of treatment weighting. **Results:** No significant differences were found in major adverse cardiovascular events (MACE—stroke, acute myocardial infarction, cardiovascular death) between the macrolide groups, and the amoxicillin/hazard ratios (HR) were azithromycin HR = 1.01, clarithromycin HR = 0.99, and roxithromycin HR = 1.02. Similarly, sensitivity analysis showed no disparities in all-cause mortality and cardiovascular death among the groups. **Conclusions:** Overall, the study revealed no evidence of increased risk of MACE, all-cause mortality, or cardiovascular death in COPD patients treated with these macrolides compared to amoxicillin over a 3-year period.

## 1. Introduction

### 1.1. Background

More than 12% of the world’s population suffers from chronic obstructive pulmonary disease (COPD) [1]. COPD is associated with both non-infectious and infectious exacerbations and thereby increases the risk of hospital admissions and death [2]. In addition, patients with COPD have a higher risk of contracting community-acquired pneumonia (CAP) compared to healthy individuals [3,4]. Pneumonia is treated with antibiotics, often β-lactam, macrolides, tetracyclines, or others [2,5]. Compared to healthy individuals, COPD patients are more often treated with antibiotics because of pneumonia and infectious exacerbations. Macrolides are a common choice to treat COPD patients presenting with atypical pneumonia, and moderate to severe pneumonia and are widely used as alternatives for patients with suspected allergies to β-lactams. Therefore, it is crucial to investigate the risk of cardiovascular events and death associated with using these antibiotics for this patient group. Given the significant reliance on these antibiotic classes, it becomes paramount to look into the potential risks they may pose, particularly concerning cardiovascular events and mortality rates. The relationship between antibiotic treatment and these adverse outcomes warrants rigorous investigation to ensure the safety and well-being of COPD patients. Understanding the implications of antibiotic use on cardiovascular health is essential, as these patients are typically more vulnerable to such risks due to their pre-existing respiratory conditions. Ensuring that the benefits of treating respiratory infections with these medications outweigh the potential hazards is a critical component of providing comprehensive care to individuals with COPD.

Several trials have investigated the short-term side effects of azithromycin, roxithromycin, and clarithromycin compared to β-lactams. Wayne et al. [6] found an increased risk for cardiovascular death and all-cause mortality for patients taking 5 or 10 days of treatment with azithromycin compared to the amoxicillin and no antibiotic groups. Svanstrom et al. [7] found in a prospective study a higher risk of cardiovascular death when taking clarithromycin compared to penicillin V. Furthermore, the U. S. Food and Drug Administration (FDA) published, at the beginning of 2018, a warning against treating patients with cardiac illnesses with clarithromycin because the CLARICOR-study group had shown an increased cardiovascular 3-year mortality (hazard ratio HR 1.42 [95% CI]) in patients treated with clarithromycin compared to placebo. The patients included in the randomized clinical trial were in a stable period of their cardiac illness (stable angina pectoris or more than 90 days since acute cardiac event).

Previous studies tend to show a higher risk for cardiovascular events with macrolides [8,9]. A few studies have been conducted on COPD patients treated with macrolides, and the results were similar [10]. However, to our knowledge, there are no studies investigating the long-term risk of cardiovascular death and cardiovascular events after being treated with azithromycin, clarithromycin, and roxithromycin for patients with COPD. Amoxicillin is a well-known antibiotic given to most patients with COPD during pneumonia; therefore, we used it as the reference for all analyses [11].

### 1.2. Objective

We aimed to determine the long-term (3 year) risk of major adverse cardiovascular events (MACE) in patients with COPD who received short treatment courses with the three most used macrolide antibiotics compared to beta-lactam antibiotic (azithromycin/roxithromycin/clarithromycin vs. amoxicillin) given for any indication.

## 2. Method

### 2.1. Data Sources and Covariates

This was a 3-year follow-up, register study using nationwide registers:

(1) The Danish Register of Chronic Obstructive Pulmonary Disease DrCOPD-Data were collected from all COPD outpatients across Denmark. Numerous beneficial variables are available. These variables include the Medical Research Council Dyspnea Scale (MRC), which measures respiratory distress; Forced Expired Volume in the first second (FEV1), a critical measure of lung function; Body Mass Index (BMI), reflecting patients’ nutritional and health status; along with the dates of outpatient visits, providing insights into healthcare utilization patterns. Additionally, demographic details such as age and gender, alongside crucial life events such as the date of death, were also captured. The Danish Register of Chronic Obstructive Pulmonary Disease (DrCOPD) is an important resource, meticulously crafted to strengthen the capacities of healthcare professionals, researchers, and policymakers in their quest to comprehend and tackle COPD with greater efficacy in the Danish context. Within this rich database lie invaluable variables, each offering a unique lens through which the complexities and severity of COPD can be discerned and categorized. The integrity and depth of this data repository are upheld through the diligent contributions of physicians and nurses operating within outpatient clinics, ensuring a robust and reliable foundation for advancing COPD care and research initiatives.
(2) The Danish National Patient Registry (DNPR)—we had access to all of the patient’s hospital admissions in Denmark registered with ICD-10. They are divided into A and B diagnoses. We used the A diagnosis to find the cardiovascular event admissions registered after each patient study entry. We used the B diagnosis to find the patient’s comorbidities. We looked at the 10-year prior baseline for the comorbidities. The Danish National Patient Registry is a comprehensive medical database that collects data on all hospital admissions and outpatient visits in Denmark, offering invaluable insights for healthcare research and policy-making by tracking patient diagnoses, treatments, and outcomes since its inception in 1977. The outpatient visits have been recorded since 1995.
(3) The Danish National Health Service Prescription Database (DNHSPD)- we obtained all of the patient’s prescriptions. Each prescription is named by its Anatomical Therapeutic Chemical ATC) classification system. The following ATC codes were used for the four groups: amoxicillin ‘J01CA04’, azithromycin ‘J01FA10’, clarithromycin ‘J01FA09’, and roxithromycin ‘J01FA06’. The Danish National Health Service Prescription Database meticulously records all prescriptions dispensed at Danish pharmacies, providing a detailed overview of medication use patterns across the population. This extensive database is instrumental in pharmaceutical research and healthcare policy development, enabling the study of prescription trends, drug safety, and adherence. Data have been registered since 2004.
(4) With the Danish National Death Registry, we can now identify the reason for death for each patient. The Danish National Death Registry is a resource that compiles information on all deaths occurring within Denmark, including causes and dates. This registry plays a vital role in epidemiological studies and public health planning, offering insights into mortality trends, life expectancy, and the impact of specific health interventions.

### 2.2. Study Design

We conducted a nationwide cohort study comparing the risk of MACE, cardiovascular death, and all-cause mortality in COPD patients treated with amoxicillin compared to treatment with azithromycin, clarithromycin, or roxithromycin. Amoxicillin was used as a reference for all analyses (flowchart).

In the DrCOPD population, all patients were registered with a COPD diagnosis from 1 January 2008 to 31 October 2017. There were 57,843 outpatients with COPD. The study entry for each patient was their first outpatient visit.

The patients were followed for three years and were censured for study outcome, end of the study period, and if they were prescribed another antibiotic than the one, they received at the study entry.

Missing values for MRC, FEV1, and BMI were handled by the last observation carried forward and amputating the median for those patients who still had a missing value.

**Figure A1:**
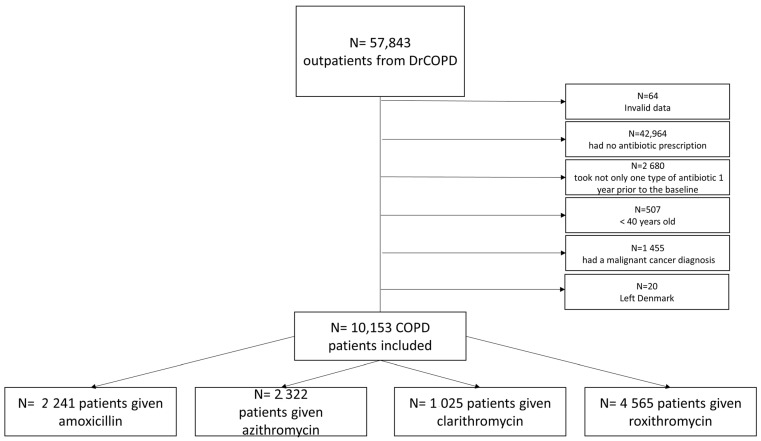
Flowchart: Study population DrCOPD.

We excluded patients with a mix of antibiotics one year prior to study entry, patients with a cancer diagnosis but not basocelullar carcinoma (registered within 10 years prior to baseline), patients who emigrated out of Denmark or disappeared from the system, and patients under the age of 40. We ended up with a study population of 10,153 outpatients with COPD (see Appendix A Figure A1).

### 2.3. Exposure to Antibiotics

To divide the population into the four antibiotic groups (clarithromycin, azithromycin, roxithromycin, and amoxicillin), we examined each patient’s antibiotic use one year before study entry by merging the population to the Danish National Health Service Prescription. Furthermore, the population was then divided into four antibiotic groups. We then followed the patients for 3 years.

### 2.4. Outcome

The primary outcome was defined as MACE (major adverse cardiovascular events). The secondary endpoint was all-cause mortality and cardiovascular death. We used the common definition of MACE: acute myocardial infarction, stroke, or cardiovascular death [12].

### 2.5. Statistical Analysis

We used the chi-square test and Wilcoxon test to compare baseline characteristics of both categorical and continuous variables, respectively. To make a time-to-event analysis, we conducted an adjusted Cox proportional hazard regression analysis. In addition, we adjusted for the following known and suspected confounders: gender, age, forced expired volume in 1 s (FEV1), smoking, prescriptions of acetylsalicylic acid one year prior to baseline, prescription of non-vitamin K antagonist oral anticoagulant (NOAC) one year prior to baseline, and some comorbidities (stroke, acute myocardial infarction, heart failure, diabetes mellitus with/without complications, and peripheral vascular disease).

In a sensitivity analysis, we used inverse probability treatment weighting (IPTW) to match the four groups, thereby avoiding confounding by indication. We matched them for the same variables as in the adjusted analysis. We then used the weighted variable in the Cox proportional hazard regression analysis. All statistical analyses were performed using SAS 9.4 (Cary, NC, USA), and R version 3.0.

### 2.6. Patient and Public Involvement

In the Steering Committee of our organization (COP:TRIN), we have patient representation (please see: [13]) to make sure that patients’ opinions and interpretations are taken seriously and can thus influence the design and conduct of trials and observational studies such as this one.

Further, we have recently, as chairs and initiators, conducted a global Delphi survey on outcomes in COPD trials, in which patients, relatives, and patient organizations were invited and participated [14].

We seek to inform patients, whenever we do nationwide observational studies, by making press releases and by discussing results with the Danish lung patient organization [15].

## 3. Results

### 3.1. Participants

The study included 10,153 subjects with chronic obstructive pulmonary disease (COPD), with specific attention paid to their antibiotic treatment regimens. The distribution of subjects across different antibiotics was 2241 (22.1%) for Amoxicillin, 2322 (22.9%) for Azithromycin, 1025 (10.1%) for Clarithromycin, and 4565 (45.0%) for Roxithromycin (see Table 1). The population had a higher proportion of females (57.5%), especially in the Roxithromycin group (59.6%).

The median percentage of the expected Forced Expiratory Volume in the first second (FEV1) was 48.0 for all subjects, indicating a moderate level of lung function impairment. The age spanned from 40 to 98 years, with a median of 69 years.

Regarding medication use, 2989 subjects (29.9%) were taking aspirin, and 2954 (29.1%) were on RAS inhibitors. Novel Oral Anticoagulants (NOAK) were used by 451 individuals (4.5%), and beta-blockers were prescribed to 2604 subjects (25.6%).

In terms of antibiotic courses taken, the majority of the cohort (8621 subjects or 85.1%) had two or fewer antibiotic courses, while 1532 subjects (15.1%) had more than two courses.

The median score on the Medical Research Council (MRC) dyspnea scale was 3.0, suggesting a moderate to severe level of breathlessness among subjects. The median Body Mass Index (BMI) was recorded as 25.0 kg/m², which falls into the higher normal range or slightly overweight category.

Comorbid conditions were also recorded, including peripheral vascular disease (11.3%), ischemic heart disease (8.9%), heart failure (14.1%), diabetes without complications (12.5%), diabetes with complications (4.0%), stroke (11.0%), renal disease (4.1%), rheumatological diseases (4.5%), and paraplegia (0.5%).

We also present an overview of the number of events in each antibiotic group (see Table 2).

#### 3.1.1. Primary Outcome

**Figure 1:**
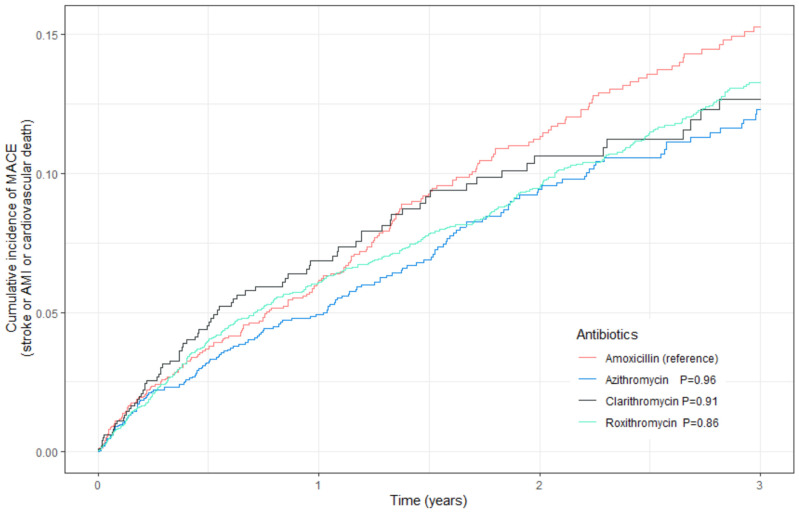
Cumulative incidence of the DrCOPD population for major adverse cardiovascular events (MACE).

Comparing the amoxicillin group with the three macrolides (clarithromycin/azithromycin/roxithromycin), we found no statistically significant difference between the groups for MACE in the adjusted Cox proportional hazard regression analysis (Azithromycin: HR = 1.01, 95% CI 0.81–1.25, *p* value = 0.96; Clarithromycin: HR = 0.99, 95% CI 0.75–1.30, *p* value = 0.91; Roxithromycin: HR = 1.02, 95% CI 0.85–1.22, *p* value = 0.86, see Figure 1 and Table 3). Using the inverse probability treatment weighting, the results were unchanged (azithromycin: HR = 0.94, 95% CI 0.76–1.16, *p* value = 0.54; clarithromycin: HR = 1.03, 95% CI 0.79–1.35, *p* value = 0.82; roxithromycin: HR = 1.00, 95% CI 0.83–1.19, *p* value = 0.97, Table 3). As a post hoc sensitivity analysis, we also combined the three macrolides and compared them to amoxicillin. The result remains unchanged for MACE (HR = 0.99, 95% CI 0.96–1.02, *p* value = 0.43).

We hypothesized that macrolides could be associated with cardiac arrhythmia and conducted a secondary analysis for the risk of atrial fibrillation for each of the groups in our study. We found no difference between the three macrolides (azithromycin: HR = 1.04, 95% CI 0.94–1.16, *p* value = 0.45; clarithromycin: HR = 0.93, 95% CI 0.81–1.06, *p* value = 0.28; roxithromycin: HR = 0.97, 95% CI 0.87–1.06, *p* value = 0.50) compared to amoxicillin.

As all-cause mortality could be a competing risk to MACE, we analyzed the risk of a combined outcome (MACE or all-cause mortality) as well as cause-specific hazards for death and MACE and found no differences between the groups (Supplementary Table S1).

We tested for interaction between the number of antibiotic prescriptions (1 vs. >1) and found no interaction (*p* = 0.60).

#### 3.1.2. Secondary Outcome

For all-cause mortality and cardiovascular death, there was no statistical difference comparing amoxicillin to azithromycin, clarithromycin, and roxithromycin in the adjusted Cox proportional hazard regression (all-cause mortality: azithromycin: HR = 1.06, 95% CI 0.94–1.19, *p* value = 0.37; clarithromycin: HR = 0.95, 95% CI 0.81–1.11, *p* value = 0.51; roxithromycin: HR = 0.98, 95% CI 0.89–1.09, *p* value = 0.70, see Table 4 and cardiovascular death for azithromycin: HR = 0.96, 95% CI 0.70–1.33, *p* value = 0.82; clarithromycin: HR = 1.16, 95% CI 0.79–1.69, *p* value = 0.45; roxithromycin: HR = 1.12, 95% CI 0.87–1.45, *p* value = 0.37, see Table 5). Using IPTW analysis, the results were similar (all-cause mortality: azithromycin: HR = 0.98, 95% CI 0.88–1.11, *p* value = 0.78 clarithromycin: HR = 0.93, 95% CI 0.80–1.08, *p* value = 0.31; roxithromycin: HR = 0.98, 95% CI 0.88–1.08, *p* value = 0.63 and cardiovascular death was azithromycin: HR = 0.87, 95% CI 0.64–1.19, *p* value = 0.40; clarithromycin: HR = 1.22, 95% CI 0.85–1.74, *p* value = 0.27; roxithromycin: HR = 1.1, 95% CI 0.85–1.40, *p* value = 0.48, Table 4 and Table 5).

## 4. Discussion

In our nationwide, registry-based cohort study of 10,513 COPD patients, we found no evidence of differences between amoxicillin and clarithromycin/azithromycin/roxithromycin regarding major adverse cardiovascular events (MACE) in both the adjusted Cox regression and IPTW analysis. Similarly, we did not observe any differences between amoxicillin and the three macrolides in terms of all-cause mortality or cardiovascular death, which were secondary outcomes in the DrCOPD population.

Previous studies have investigated the use of azithromycin, roxithromycin, or gatifloxacin in patients with coronary heart disease [16,17,18]. The overall consensus from these studies is that antibiotics do not significantly impact cardiovascular events [19,20]. Although our study focused on COPD patients, our results align with those previous findings. However, it is worth noting that the CLARI-COR study, which examined stable heart disease patients, did find a higher risk of cardiovascular events and all-cause mortality with 14 days of clarithromycin compared to placebo.

It is possible that clarithromycin/azithromycin/roxithromycin may increase the risk of MACE, all-cause mortality, and cardiovascular death in COPD patients when compared to placebo. However, it is important to consider that our study evaluated the three macrolides individually versus amoxicillin, not versus placebo. Furthermore, the CLARICOR study did not administer clarithromycin based on a clinical indication, whereas the patients who received macrolides in our study had relevant symptoms. A potential explanation for the lack of excess risk of death from all causes and myocardial infarction in the clarithromycin-treated group, as documented in the CLARICOR trial, could be that our patients had a dominant competing risk of death from pulmonary causes that outweighed the risk of death from cardiac causes. This is an important distinction from the patients included in the CLARICOR trial, who likely did not have a significant risk of death from lung disease. COPD patients have a higher occurrence of respiratory infections compared to the general population, leading to increased macrolide usage. Additionally, when comparing them to age-matched individuals in the background population, they face a greater risk of major adverse cardiovascular events (MACE). Despite acknowledging competing risks, it is crucial to report the associated risk for the reasons mentioned.

To date, only a few studies have examined macrolide use in COPD patients despite their considerable risk of cardiovascular events. In 2013, Schembri et al. conducted a prospective cohort study [10] investigating the association of clarithromycin with cardiovascular events in patients with acute exacerbation of COPD (AECOPD) or community-acquired pneumonia (CAP). Their findings indicated an increased risk of cardiovascular events and acute coronary syndrome associated with clarithromycin for AECOPD (HR 1.50, 95% CI 1.13–1.97) and CAP (HR 1.67, 95% CI 1.04–2.68). Although these results differ from ours, it is important to note that Schembri et al. examined the association between macrolides and MACE for acute diseases (AECOPD and CAP), while our study included stable outpatients with COPD. Therefore, our patients were in a stable phase, while Schembri et al. possibly included “frequent exacerbators” in their study of COPD patients. Additionally, their follow-up period was one year, whereas we followed our patients for three years. However, their findings regarding all-cause mortality were similar to ours, indicating that macrolides did not increase the risk of death from any cause. We obtained similar results for mortality, but we did not find any differences between the groups for MACE.

Another study by Wong et al. [21] compared clarithromycin to amoxicillin. They found that the current use of clarithromycin was associated with an increased risk of arrhythmia, myocardial infarction, and cardiac mortality. However, they did not observe any association with long-term cardiovascular risks in the Hong Kong population. This study is similar to ours as it compares the same antibiotics. However, they examined a considerably healthier population by including all patients over 18 years old who had a prescription for either amoxicillin or clarithromycin.

### Strengths and Limitations

Our study is distinguished by several significant strengths that contribute to the robustness of our conclusions. Firstly, the utilization of comprehensive databases such as DrCOPD has been instrumental in our research, providing us with complete data on every patient. This access to extensive patient information has allowed us to define our study population with great precision, thereby ensuring a larger sample size.

Another notable aspect of our study is the categorization of patients into four distinct groups based on their antibiotic treatment, with careful measures taken to ensure that patients did not receive any of the other studied antibiotics during the observation period. This strict censure adds a layer of rigor to our study design, ensuring that the outcomes observed are specifically attributable to the antibiotics under study, thereby minimizing potential confounding factors.

Furthermore, our application of inverse probability treatment weighting (IPTW) in the analysis is a testament to the methodological rigor of our study. IPTW has allowed us to adjust for baseline differences between the groups without the need to exclude any patients from the analysis. This method not only helps in controlling for confounding factors but also in preserving the integrity and size of our study population, which is crucial for the reliability of our findings.

Importantly, our study is to our knowledge the first to compare the use of amoxicillin with three different macrolides in the context of COPD treatment. Previous research has often compared antibiotic use with non-use, which does not account for the confounding effect of the underlying infections necessitating antibiotic therapy. Our approach, therefore, provides a more nuanced understanding of the relative safety of these antibiotics, potentially informing clinical decisions in the management of COPD patients.

Despite these strengths, our study is not without limitations. The lack of detailed information on the specific indications for antibiotic prescriptions is a notable drawback, as it introduces an element of uncertainty regarding the comparability of the treatment groups. Nevertheless, physicians might preferentially prescribe amoxicillin over other antibiotics due to an awareness of increased cardiovascular risks associated with other agents. Additionally, the inherent differences between the groups, despite our best efforts at adjustment, and the retrospective nature of our study introduce potential biases that must be considered when interpreting our findings. Moreover, the challenge of comparing our results with those from studies involving different patient populations highlights the complexity of conducting and interpreting clinical research. Lastly, the possibility of residual confounding, inherent in observational studies, cannot be entirely ruled out, underscoring the need for cautious interpretation of our results.

In summary, while our study makes significant contributions to the understanding of the cardiovascular safety of macrolide antibiotics in COPD patients, it also highlights the intricate balance between methodological strengths and the inevitable limitations of observational research. Our findings pave the way for future studies to further explore this important area, building on the foundation we have established to enhance the care of COPD patients.

## 5. Conclusions

In conclusion, we found no evidence of a difference in the risk of MACE or death from all causes in a 3-year follow-up period between COPD patients who were prescribed one of the following antibiotics: amoxicillin, clarithromycin, azithromycin, and roxithromycin. This is reassuring since these antibiotics are commonly prescribed in this patient group. Additional research is necessary to draw any definitive conclusions.

## 6. Future Perspectives

An intriguing aspect that warrants further exploration within our research is the hypothesis surrounding Chlamydia pneumoniae and its potential role in exacerbating the risk of systemic inflammation, which can contribute to the development of atherosclerosis. This hypothesis is grounded in the concept that infections, particularly those caused by Chlamydia pneumoniae, might not only trigger an immediate inflammatory response but could also have long-term implications for cardiovascular health by promoting the formation of atherosclerotic plaques.

In the introductory section of our study, we briefly touched upon existing literature where researchers have conducted post-mortem examinations and discovered the presence of Chlamydia pneumoniae within atherosclerotic plaques. This finding suggests a possible link between chronic infections and the pathogenesis of atherosclerosis, underscoring the complex interplay between infectious agents and cardiovascular diseases.

Given the potential implications of Chlamydia pneumoniae in the context of cardiovascular health, it becomes imperative to look deeper into how this infection might influence the risk of developing cardiovascular diseases. Furthermore, the role of macrolide antibiotics in this equation is particularly noteworthy. Macrolides, known for their anti-inflammatory and antimicrobial properties, could potentially offer a dual therapeutic benefit by not only addressing the infectious component but also mitigating the associated inflammatory processes that contribute to atherosclerosis.

Therefore, a more nuanced investigation into the association between Chlamydia pneumoniae infection and cardiovascular diseases, with a specific focus on the mediating role of macrolide antibiotics, could provide valuable insights. Such research could unravel the mechanisms through which chronic infections influence cardiovascular risk and assess the therapeutic potential of macrolides in disrupting this link. By expanding our understanding in this area, we can pave the way for more targeted interventions that address the infectious and inflammatory dimensions of cardiovascular diseases, ultimately contributing to better management strategies and improved patient outcomes in the context of cardiovascular health.

## 7. Ethical Considerations

The information utilized in this study was sourced from routine patient care processes, ensuring minimal disruption or discomfort to the individuals involved. The confidentiality of this data was maintained with the highest level of integrity, adhering strictly to legal standards for data protection. This approach not only safeguards patient privacy but also contributes to the broader objective of enhancing healthcare for individuals with Chronic Obstructive Pulmonary Disease (COPD), underscoring the importance of such research in the realm of public health. In accordance with Danish legislation, the use of registry data for scholarly research is permissible, provided that specific conditions are fulfilled. This legal framework facilitates the ethical use of patient data for academic inquiries, enabling researchers to contribute valuable insights to the field while ensuring that patient rights and privacy are protected. This meticulous adherence to ethical and legal standards reinforces the credibility of the research and its potential to inform and improve COPD patient care practices.

## Figures and Tables

**Table 1:** Baseline characteristics: DrCOPD population.

	All	Amoxicillin	Azithromycin	Clarithromycin	Roxithromycin
**Number of subjects, *n* (%)**	10,153 (100.0)	2241 (22.1)	2322 (22.9)	1025 (10.1)	4565 (45.0)
**Females, *n* (%)**	5843 (57.5)	1158 (51.7)	1366 (58.8)	596 (58.1)	2723 (59.6)
**FEV1, % of expected, median (IQR)**	48.0 (35.0–63.0)	47.0 (34.0–62.0)	45.0 (32.0–60.0)	49.0 (36.0–63.0)	50.0 (36.0–64.0)
**Age**	69.69 (40–98)	70.85 (40–98)	68.96 (40–92)	69.39 (40–93)	69.55 (40–96)
**Asprin**	2989	722	646	305	1316
**Renin-Angiotension-System (RAS)-inhibitors**	2954	698	604	300	1352
**Novel oral anticoagulants (NOAC)**	451	98	115	47	191
**Beta-blockers, *n* (%)**	2604	675	523	258	1148
**Amount prescriptions of antibiotic courses:**					
**≤2**	8621	1998	1677	925	4021
**>2**	1532	243	645	100	544
**MRC, median (IQR)**	3.0 (2.0–4.0)	3.0 (2.0–4.0)	3.0 (2.0–4.0)	3.0 (2.0–4.0)	3.0 (2.0–4.0)
**BMI, *n* (%), kg/m²**	25.0 (21.0–29.0)	25.0 (21.0–29.0)	24.8 (21.0–28.4)	25.0 (21.2–29.0)	25.0 (22.0–29.0)
**Peripheral vascular disease, *n* (%)**	1149 (11.3)	284 (12.7)	233 (10.0)	118 (11.5)	514 (11.3)
**Ischaemic heart disease, *n* (%)**	902 (8.9)	241 (10.8)	186 (8.0)	98 (9.6)	377 (8.3)
**Heart failure, *n* (%)**	1430 (14.1)	375 (16.7)	285 (12.3)	139 (13.6)	631 (13.8)
**Diabetes without complications, *n* (%)**	1266 (12.5)	278 (12.4)	261 (11.2)	132 (12.9)	595 (13.0)
**Diabetes with complications, *n* (%)**	411 (4.0)	92 (4.1)	72 (3.1)	45 (4.4)	202 (4.4)
**Stroke, *n* (%)**	1115 (11.0)	266 (11.9)	214 (9.2)	126 (12.3)	509 (11.2)
**Renal disease, *n* (%)**	415 (4.1)	115 (5.1)	74 (3.2)	52 (5.1)	174 (3.8)
**Rheumatological disease, *n* (%)**	460 (4.5)	99 (4.4)	108 (4.7)	50 (4.9)	203 (4.4)
**Paraplegia, *n* (%)**	46 (0.5)	12 (0.5)	12 (0.5)	7 (0.7)	15 (0.3)

Abbreviations: FEV1: forced expiratory volume in the first second, Medical Research Council dyspnea, BMI: Body Mass Index (kg/m^2^).

**Table 2:** Number of events for each endpoint for each antibiotic.

Antibiotic Groups	End of Follow-Up *N* (%)	Stroke *N* (%)	AMI ** N (%)	Cardiovascular Death *N* (%)	Cause of Death * N (%)	Antibiotic Switch *N* (%)	Total (*n*)
**Amoxicillin**	1092 (48.73)	74 (3.30)	37 (1.65)	84 (3.75)	502 (22.40)	452 (20.17)	2241
**Azithromycin**	1329 (57.24)	58 (2.50)	41 (1.77)	63 (2.71)	473 (20.37)	358 (15.42)	2322
**Clarithromycin**	553 (53.95)	29 (2.83)	13 (1.27)	38 (3.71)	183 (17.85)	209 (20.39)	1025
**Roxithromycin**	2568 (56.25)	128 (2.80)	78 (1.71)	174 (3.81)	1007 (22.06)	610 (13.36)	4565
**Total**	5542 (54.58)	289 (2.85)	169 (1.66)	359 (3.54)	2165 (21.32)	1629 (16.04)	10,153

* Other causes than cardiovascular death: main analysis. ** Acute myocardial infarction.

**Table 3:** Risk of MACE comparing the amoxicillin group to the azithromycin, clarithromycin, and roxithromycin group for the DrCOPD population (adjusted and IPTW *).

		Adjusted			IPTW	
Treatment Groups	Hazard Ratio	95% Confidential Interval	*p* Value	Hazard Ratio	95% Confidential Interval	*p* Value
Azithromycin	1.01	0.81–1.25	0.96	0.94	0.76–1.16	0.54
Clarithromycin	0.99	0.75–1.30	0.91	1.03	0.79–1.35	0.82
Roxithromycin	1.02	0.85–1.22	0.86	1.00	0.83–1.19	0.97
Amoxicillin	1.00	ref	ref	1.00	ref	ref

* inverse probability treatment weighting.

**Table 4:** Risk of all-cause mortality comparing the amoxicillin group to the clarithromycin, azithromycin, and roxithromycin group for the DrCOPD population (adjusted and IPTW *).

		Adjusted			IPTW	
Treatment Groups	Hazard Ratio	95% Confidential Interval	*p* Value	Hazard Ratio	95% Confidential Interval	*p* Value
Azithromycin	1.06	0.94–1.19	0.37	0.98	0.88–1.11	0.78
Clarithromycin	0.95	0.81–1.11	0.51	0.93	0.80–1.08	0.31
Roxithromycin	0.98	0.89–1.09	0.70	0.98	0.88–1.08	0.63
Amoxicillin	1.00	ref	ref	1.00	ref	ref

* inverse probability treatment weighting.

**Table 5:** Risk of cardiovascular death comparing the amoxicillin group to the azithromycin, clarithromycin, and roxithromycin group for the DrCOPD population (adjusted and IPTW *).

		Adjusted			IPTW	
Treatment Groups	Hazard Ratio	95% Confidential Interval	*p* Value	Hazard Ratio	95% Confidential Interval	*p* Value
Azithromycin	0.96	0.70–1.33	0.82	0.87	0.64–1.19	0.40
Clarithromycin	1.16	0.79–1.69	0.45	1.22	0.85–1.74	0.27
Roxithromycin	1.12	0.87–1.45	0.37	1.1	0.85–1.40	0.48
Amoxicillin	1.00	ref	ref	1.00	ref	Ref

* inverse probability treatment weighting.

## Data Availability

The data presented in this study are available on request from the corresponding author (accurately indicate status).

## Supplementary Materials

The following supporting information can be downloaded at: https://www.mdpi.com/article/10.3390/jcm13071987/s1, Table S1: Post hoc sensitivity analysis: Risk of MACE comparing the amoxicillin group to the azithromycin, clarithromycin and roxithromycin group for DrCOPD population (adjusted) with a 1-year follow-up; Table S2: Competing risk analysis of primary outcome comparing the amoxicillin group to the azithromycin, clarithromycin and roxithromycin group for DrCOPD population. Risk of combined outcome “MACE or death by any cause” as well as cause specific hazards for MACE and death, in which the opposite outcome is right censored.
