# Supplementary material for: Risk of Mortality and Cardiovascular Events in Patients with Chronic Obstructive Pulmonary Disease Treated with Azithromycin, Roxithromycin, Clarithromycin, and Amoxicillin
Source: J Clin Med. 2024 Mar 29;13(7):1987. doi: 10.3390/jcm13071987 (PMC11013008; doi:10.3390/jcm13071987)
Supplement: Supplementary file 1 [file jcm-13-01987-s001.zip › jcm-2896212-supplementary.pdf]

Supplementary

	Adjusted		
**Treatment groups**	**Hazard Ratio**	**95% Confidential interval**	**P value**
Azithromycin	0.98	0.73-1.32	0.90
Clarithromycin	1.23	0.87-1.74	0.23
Roxithromycin	1.12	0.88-1.43	0.35
Amoxicillin	1.00	ref	ref

Table S1. Post hoc sensitivity analysis: Risk of MACE comparing the amoxicillin group to the azithromycin, clarithromycin and roxithromycin group for DrCOPD population (adjusted) with a 1-year follow-up: Abbreviations: DrCOPD: Danish registry of COPD, IPTW: Inverse Probability of Treatment Weighting, MACE: Major Adverse Cardiac Event

		Adjusted		
**Treatment groups**		**Hazard Ratio**	**95% Confidential interval**	**P value**
MACE or death by any cause				
	Azithromycin	1.06	0.94-1.18	0.36
	Clarithromycin	0.93	0.80-1.08	0.34
	Roxithromycin	0.99	0.90-1.08	0.76
	Amoxicillin	1.00	ref	ref
Cause specific risk of MACE				
	Azithromycin	1.01	0.81-1.25	0.96
	Clarithromycin	0.99	0.75-1.30	0.91
	Roxithromycin	1.02	0.86-1.22	0.86
	Amoxicillin	1.00	ref	ref
Cause specific risk of death by any cause				
	Azithromycin	1.07	0.94-1.23	0.29
	Clarithromycin	0.91	0.76-1.09	0.30
	Roxithromycin	0.97	0.87-1.09	0.64
	Amoxicillin	1.00	ref	ref

Table S2. Competing risk analysis of primary outcome comparing the amoxicillin group to the azithromycin, clarithromycin and roxithromycin group for DrCOPD population. Risk of combined outcome "MACE or death by any cause" as well as cause specific hazards for MACE and death, in which the opposite outcome is right censored.
